# *Pseudomonas aeruginosa* in *Musca domestica* L.: Temporospatial Examination of Bacteria Population Dynamics and House Fly Antimicrobial Responses

**DOI:** 10.1371/journal.pone.0079224

**Published:** 2013-11-18

**Authors:** Chester Joyner, Mary Katherine Mills, Dana Nayduch

**Affiliations:** Department of Biology, Georgia Southern University, Statesboro, Georgia, United States of America; Rockefeller University, United States of America

## Abstract

House flies associate with microbes throughout their life history. Bacteria ingested by adult flies enter the alimentary canal and face a hostile environment including antimicrobial defenses. Because the outcome of this interaction impacts bacterial survival and dissemination, our primary objective was to understand the temporospatial dynamics of fly-bacteria associations. We concurrently examined the temporospatial fate of GFP-expressing *Pseudomonas aeruginosa* (GFP-*P. aeruginosa*) in the house fly alimentary canal along with antimicrobial peptide (AMP) expression. Motile, viable GFP-*P. aeruginosa* were found in all regions of the alimentary canal and were culturable throughout the observation period (2–24 h). A significant decrease in recoverable bacteria occurred between 2 and12 h, followed by an increase between 12 and 24 h. qRT-PCR analysis showed expression of the AMPs *cecropin*, *diptericin*, and *defensin* both locally (gut) and systemically. Furthermore, mRNA of all AMPs were expressed throughout gut tissues, with some tissue-specific temporal variation. Interestingly, fluctuation in recoverable *P. aeruginosa* was associated with AMP protein expression in the gut (immunofluorescent signal detection), but not with mRNA (qRTPCR). In regards to vector competence, flies excreted GFP-*P. aeruginosa* throughout the 24 h period, serving as both reservoirs and disseminators of this bacterium. Collectively, our data show flies can harbor and disseminate *P. aeruginosa*, and that the interactions of fly defenses with bacteria can influence vector competence.

## Introduction

Since larvae require bacteria for survival and development [1], house flies associate with septic substrates teeming with microbes throughout their entire life history [2]. In addition, house flies are synanthropic insects that live, breed, and feed in close contact to humans. House flies indiscriminately move between these niches and, therefore, have been implicated in the transmission of over 65 human diseases caused by microbes such as *Salmonella*, *Escherichia coli*, *Shigella*, *Streptococcus*, *Staphylococcus*, and *Pseudomonas*, especially in developing countries where sanitation practices are poor [3]–[11].

House flies transmit bacteria both mechanically, via contaminated mouthparts and legs, and biologically, via ingestion of microbes and excretion in vomit or feces. Food, including ingested bacteria, usually is first stored in the crop and predigested by salivary carbohydrases before entering the gut [12]. However, the fly often will expel crop contents to help liquefy subsequent meals and, therein, transmits bacteria by regurgitation. Ingested materials eventually enter the alimentary canal by passing through the proventriculus to the midgut, and at that point cannot be regurgitated. The midgut epithelium is protected by a type-II peritrophic matrix (PM), which serves as a size-exclusive physical barrier to microbes [13], [14]. Although the PM is impermeable to bacteria, large molecules including digestive enzymes and antimicrobial effectors produced by the gut epithelium easily traverse and act on targets within the lumen [15]. Transmission of bacteria in fly feces therefore requires that they survive these harsh midgut conditions, including entrapment in the PM, digestive processes and antimicrobial defenses, as they move by peristalsis towards the rectum for expulsion.

Part of the dipteran antimicrobial defense is the humoral immune response, whose end products are effector molecules that have a range of functions from protecting flies from pathogen assault to bacterial population control in the gut. *Drosophila melanogaster*, which also resides in microbe-rich habitats, recognize gram-negative bacteria (and some gram-positive bacilli) when diaminopimelic acid-type peptidoglycan (DAP-PGN) fragments released from lysed or dividing microbes bind transmembrane peptidoglycan recognition receptors (PGRPs) on epithelial cells and other tissues [16]–[20]. Immune cascades, such as the Imd pathway, are then induced and result in the transcriptional activation of antimicrobial peptides (AMPs) that have microbicidal and microbistatic activities [21].

*Pseudomonas aeruginosa* is a pan-antibiotic resistant pathogen of humans and is commonly isolated from wild-caught house flies [10], [22]. In this study, our aim was to assess the temporospatial dynamics of fly-microbe interactions from (1) the microbe perspective, by determining the temporospatial fate of GFP-expressing bacteria (GFP-*P. aeruginosa*) including viability, location in the alimentary canal, population dynamics, and excretion and (2) the house fly perspective, by concurrently examining the spatial expression of selected AMPs (cecropin, diptericin and defensin). We determined that GFP-*P. aeruginosa* persisted in the entire house fly alimentary canal for up to 24 h post-ingestion (PI), and was excreted throughout the observation period. As soon as 2 h PI, AMP mRNAs were upregulated in response to bacteria both systemically (e.g. fat body) and locally in the alimentary canal. Interestingly, a decline in the amount of GFP-*P. aeruginosa* recovered from flies was preceded by local (gut) expression of AMP protein. This study provided a unique view of the dynamic interactions between house flies and bacteria and revealed that these associations influence bacterial persistence, survival and, ultimately, transmission potential.

## Materials and Methods

### Bacteria Culture

*Pseudomonas aeruginosa* strain PAK*gfp* (GFP-*P. aeruginosa*) was obtained from Dr. Marina Ulanova at Lakehead University [23]. For all experiments, GFP-*P. aeruginosa* cultures were maintained on tryptic soy agar (TSA; Fisher Scientific, Atlanta, GA) with 30 µg/ µl gentamicin. Before all fly feedings, described below, bacteria were grown in brain heart infusion broth (BHI; Fisher Scientific) with gentamicin (30 µg/ µl) at 37°C to an OD_600_ near 0.1, which is ≈10^8^ colony forming units (CFU) per ml.

### House Fly Rearing and Treatment

Adult house flies from the Georgia Southern University colony were maintained as previously described [24], and pupae were removed for eclosion into sterile petri dishes. Flies that emerged were not axenic but were confirmed free of the test strain of bacteria by culturing of a subset of individuals from each biological replicate onto TSA before each experiment. Prior to use in experiments, freshly eclosed, mixed-sex adult house flies were individually-housed in 50-ml glass jars and fed a single sterile 5 µl droplet of fly food (10% w/v) followed by fasting for 8–12 h at 25°C. After being maintained at 30°C for 2 h to induce feeding, each house fly was then fed a 2 µl droplet of GFP-*P. aeruginosa* culture. The amount of bacteria in the droplet was determined by serial dilution and culture on TSA-gentamicin as described above.

### Localization of GFP-*P. Aeruginosa* in the House Fly Alimentary Canal

Flies (n = 20 in each of 3 independent biological replicates) were individually-housed and fed GFP-*P. aeruginosa* as described above (OD_600_ = 0.140; mean 2.12×10^5^ CFU/fly; SD = 8.36×10^4^). At 2, 6, 10, 12, and 24 h PI, flies (n = 4/replicate) were killed and aseptically dissected in sterile phosphate buffer saline (PBS) to remove the intact alimentary canal (i.e. proventriculus, crop, midgut, hindgut, and rectum). Tissues were examined for the location, presence, motility, and viability of GFP-*P. aeruginosa* using epifluorescent and light microscopy. Digital photographs were captured with a Leica DFC420 digital camera on a Laborlux 12 microscope (Leitz, Germany).

### Recovery of Viable GFP-*P. Aeruginosa* from whole House Flies

Flies (n = 20 in each of 3 independent biological replicates) were individually housed and fed GFP-*P. aeruginosa* as described above (OD_600_ = 0.111; mean 3.60×10^5^ CFU/fly; SD = 1.75×10^5^). At 2, 6, 10, 12, and 24 h PI, flies (n = 4) were sacrificed then surface sanitized in 70% ethanol followed by 10% Clorox® bleach for 10 min each. After drying, individual flies were macerated in 500 µl sterile PBS, and homogenate was serially diluted and cultured overnight at 37°C on TSA-gentamicin to enumerate the surviving CFU in each fly. Changes in the number of recoverable GFP-*P. aeruginosa* were calculated as the difference between the CFU recovered at each time point and the CFU fed to each fly in that replicate. Only agar plates with 30 to 300 CFU were included in calculations (i.e., 169 out of 180 plates observed were included) and subsequent statistical analyses. Kruskall-Wallis and Mann-Whitney U tests were used to analyze the overall change in bacterial survival over time and differences between time points, respectively (JMP® v. 8.0.1; SAS Institute Inc., Cary, NC, USA).

### Recovery of Viable GFP-*P. Aeruginosa* from House Fly Excreta

Flies (n = 20 in each of 3 independent biological replicates) were individually-housed and fed GFP-*P. aeruginosa* as described above (OD_600_ = 0.127; mean 2.02×10^5^ CFU/fly; SD = 6.30×10^4^). After feeding, flies were immobilized by chilling at 4°C and transferred to individual 35×10 mm sterile Petri dishes (Fisher Scientific). At 2, 6, 10, 12, and 24 h PI, flies (n = 4) were euthanized and each dish was washed with 1 ml of sterile PBS. Each wash was individually cultured on selective media as described above in order to enumerate GFP-*P. aeruginosa* shed in excreta during those time intervals (from 0 h to each time point listed above).

### Local and Systemic AMP Gene Expression in House Flies

Flies (n = 25 in each of 3 independent biological replicates) were housed and fed bacteria as described above (OD_600_ = 0.145; mean 3.41×10^5^ CFU/fly; SD = 5.77×10^3^). Flies (n = 5) were aseptically dissected at 2, 6, 10, 12, and 24 h PI to obtain intact digestive tracts (i.e. crop, proventriculus, midgut, hindgut, and rectum) and carcasses (i.e. head, appendages, salivary glands, fat body, and all other tissues in the carcass). Calibrator state flies (n = 4) were fed BHI with gentamicin (30 µg/ µl) and dissected at 2 h PI as above. Tissues were homogenized in Trizol® reagent (Life Technologies, Carlsbad, CA, U.S.A) and RNA was extracted and purified using an Ambion® Ribopure™ Kit (Life Technologies) following the manufacturer’s protocol. RNA (1 µg/reaction) was reverse transcribed using the Quantitect® Reverse Transcription Kit (Qiagen, Valencia, CA). Individual qRT-PCR reactions were performed in triplicate using the RealMasterMix SyBR ROX kit (5-PRIME) with sense and antisense primers (Table S1; 500 nM each) of target genes, *diptericin* (*dpt*), *cecropin* (*cec*), and *defensin* (*def*), and the reference gene, *ribosomal protein s18* (*rps18*), and with 1∶10 diluted cDNA template. Reactions containing all components except cDNA template served as negative controls. Amplification was performed under the following conditions in a realplex^2^ mastercycler (Eppendorf North America, Hauppauge, NY): 2 min at 95°C, 32 cycles of 20 s at 61°C, 15 s at 68°C, 15 s at 95°C, and a final extension for 2 min at 68°C. Threshold cycles (C_T_) for each reaction were collected and absolute expression ratios were determined [25]. To compare AMP expression in bacteria-fed flies to the calibrator state, a pairwise fixed allocation randomization test with at least 10,000 randomizations was performed using REST-MCS®. Absolute expression ratios were pooled within time-point by gene and normality assessed using a Shapiro-Wilk analysis. Kruskall-Wallis and Wilcoxon analyses were performed using JMP 9® v. 8.0.1 (SAS Institute Inc.) to compare (1) the effect of time within AMP and body region and (2) the effect of body region within AMP and time.

### Tissue-specific AMP Gene Expression in the House Fly Alimentary Canal

Flies (n = 30 in each of 2 parallel biological replicates) were housed and fed bacteria as described above (OD_600_ = 0.1; mean 6.6×10^4^ CFU/fly). Individual alimentary canal tissues (proventriculus, crop, midgut, hindgut) were dissected from flies (n = 10) at 2, 6, and 12 h PI and pooled for RNA extraction. cDNA synthesis and qRT-PCR was performed as described above. Tissues from newly-emerged, unfed adult house flies (n = 3) served as calibrator state. C_T_ values and absolute expression ratios were determined as above, and log_10_ transformed for use in statistical analyses. Since the dataset was normally distributed, a one-way analysis of variance (ANOVA) with Tukey-Kramer post hoc test was used to determine (1) the effect of time within AMP and tissue and (2) the effect of tissue within AMP and time.

### Immunofluorescent Detection of Antimicrobial Peptides in the House Fly Alimentary Canal

Flies (n = 12 in each of 3 biological replicates) were housed and fed bacteria as described above (OD_600_ = 0.135; mean 1.05×10^5^ CFU/fly; SD = 3.37×10^4^). At 4, 6, and 8 h PI, intact house fly alimentary canals (n = 4) were removed and fixed in a 4% paraformaldehyde solution for 2 h. As a biological control, house flies (n = 10) were fed 2 µl BHI broth with gentamicin (30 µg/ µl) and dissected at 5 h PI as described above. Tissues were dehydrated using a series of alcohol washes (50%–100%) and cleared with Citrosolv™ (Fisher Scientific). Tissues from each replicate were pooled by time point and embedded into Paraplast® plus Tissue Embedding Medium (Fisher Scientific). Five micron serial sections were affixed to slides (Superfrost®, Fisher Scientific), rehydrated with a reverse alcohol series, and blocked for 1 h in StartingBlock™ T20 Blocking Buffer (Thermoscientific, Atlanta, GA, USA). Tissues were incubated overnight at room temperature with custom primary polyclonal antibodies (Genscript, Piscataway, New Jersey, USA) diluted in StartingBlock™ T20 Blocking Buffer (Thermoscientific) at the following concentrations: rabbit anti-Cecropin (20 µg/ml), chicken anti-Defensin (5 µg/ml), or mouse anti-Diptericin (6.69 µg/ml). Tissue sections were washed twice in PBS (pH = 7.4) for 5 min followed by incubation in secondary Alexa Fluor® (Invitrogen, Grand Island, New York, USA) fluorescent antibodies (2 µg/ml) overnight at room temperature: Alexa Fluor® 568 goat anti-rabbit, Alexa Fluor® 488 goat anti-chicken, or Alexa Fluor® 488 goat anti-mouse. Two slides from each time-point were incubated with secondary antibodies only as a technical control. Tissue sections were mounted with ProLong Gold© antifade reagent (Invitrogen) containing DAPI nuclear stain. Immunofluorescence was visualized using a Laborlux12 microscope (Leitz, Germany) equipped with appropriate bandpass filters and a Leica DFC420 microscope camera (Leica Microsystems, Inc., Buffalo Grove, IL, USA).

## Results and Discussion

### Viable, Motile GFP-*P. Aeruginosa* Persisted and Proliferated in the House Fly Alimentary Canal

**Figure 1:**
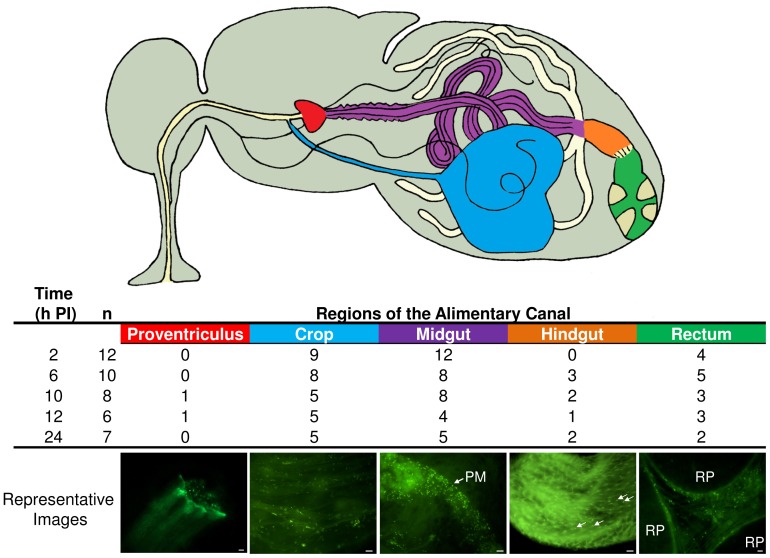
GFP-*P. aeruginosa* were found throughout the house fly alimentary canal up to 24 h post-ingestion (PI). House flies (n = 20 in each of three replicates) were fed an average of 2.12×10^5^ CFU (SD = 8.36×10^4^) bacteria. At 2, 6, 10, 12, and 24 h PI, flies (n = 4 per replicate) were dissected to remove the entire gut, and the location and viability of GFP-*P. aeruginosa* was assessed by epifluorescent microscopy. The number (“n”) of house flies observed to have viable GFP-*P. aeruginosa* is indicated (of n = 12 total flies observed/time point). Column headings of alimentary canal regions are color-coded to correspond to the same colors on the line drawing of the house fly anatomy, and the number of flies observed to have bacteria in those locations is indicated. Representative images of GFP-*P. aeruginosa* are shown, and bacteria are green rods and/or indicated by arrows. For more images, refer to Figure S1. PM, peritrophic matrix; RP, rectal pad. Scale bar = 10 µm in all images.

As soon as 2 h PI and across the entire 24 h observation period, we observed motile GFP-*P. aeruginosa* throughout the alimentary canal from crop to rectum in the majority of flies that were examined (Fig. 1; Fig. S1). In the midgut, bacteria were confined within the inner PM as has been shown with other bacterial species in previous studies [24], [26]–[28], [30]. Notably, GFP-*P. aeruginosa* did not appear to be immobilized or adhered to the inner PM as has been reported with gram-negative species such as *A. hydrophila* [26]. Instead, highly motile GFP-*P. aeruginosa* were observed moving freely in the lumen. This is similar to observations of *A. caviae,* which maintained motility in the house fly gut and, consequently, persisted for several days [27], [28]. In stark contrast, nonmotile species of bacteria such as *S. aureus* or immobilized species such as *A. hydrophila* quickly became eliminated from house flies via lysis and excretion [24], [26]. We speculate that the ability of *P. aeruginosa* to maintain directional motility enables bacteria to resist peristalsis and elimination by excretion and, therefore, facilitates persistence in the house fly gut. Further, motile bacteria also could chemotactically avoid unfavorable gut conditions or local immune responses, which consequently enhances the chance of survival [29]. Future investigations should aim to (1) elucidate factors that preserve or impair bacterial motility in the house fly gut, (2) understand how motility influences persistence and elimination, and (3) explore how these processes ultimately impact vector competence.

**Figure 2:**
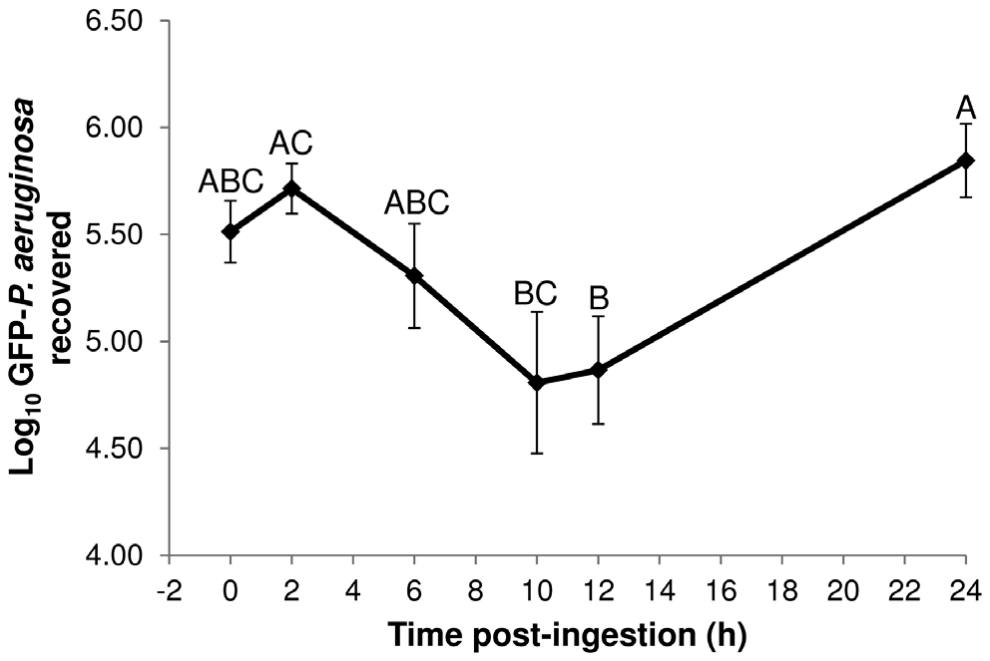
GFP-*P. aeruginosa* persisted and proliferated within the house fly. House flies (n = 20 in each of three replicates) were fed an average of 3.60×10^5^ CFU (SD = 1.75×10^5^) bacteria. At 2, 6, 10, 12, and 24 h post-ingestion, GFP-*P. aeruginosa* were enumerated from whole flies (n = 4 per replicate) by culture on selective media. Details on culture methods and statistical analysis are in the text. Different letters represent significant differences between mean CFUs recovered from flies (P≤0.04), and error bars are standard error.

After qualitatively observing the temporospatial fate of GFP-*P. aeruginosa* in the house fly gut, we subsequently quantified bacterial population dynamics by culturing GFP-*P. aeruginosa* from whole flies over 24 h post-ingestion. Numbers of GFP-*P. aeruginosa* initially decreased in flies between 2 and 12 h PI but subsequently increased between 12 and 24 h PI (Fig. 2; P≤0.04). Interestingly, although the GFP-*P. aeruginosa* population had fluctuated, the number of bacteria in house flies never exceeded 10^5^ CFU. Other species of bacteria such as *A. hydrophila*, *A. caviae* and *Enterococcus faecalis* [26], [27], [30] also replicate within the house fly while species such as *S. aureus* [24] do not. The factors that allow some bacteria species to proliferate or limit others remain to be determined, but probably involve processes in addition to motility. Nonetheless, because GFP-*P. aeruginosa* not only persisted, but also proliferated, in house flies, long-term transmission and dissemination potential is possible. Future studies will extend both qualitative (microscopy) and quantitative (culture) experiments beyond 24 h to assess the long term fate of bacteria in flies, and to determine whether the peak mean CFU we enumerated (∼10^5^ CFU) is the maximum sustainable number that can persist in the house fly gut.

Because GFP-*P. aeruginosa* persisted and proliferated in flies, we additionally assessed transmission potential by monitoring excretion of bacteria within several time intervals in the 24 h observation period. While relatively few house flies excreted bacteria in each time interval (incidence ranged from 16.7–41.7% of flies), flies that did shed GFP-*P. aeruginosa* had excreted from as few as 470 to as many as 2.03×10^5^ CFU (Table 1). While there appeared to be no direct correlation between the amount excreted and the length of the time interval, flies excreted the largest amount of bacteria (mean 9.98×10^4^ CFU) between 0 and 12 h PI, which incidentally was the same time interval after which we noted a significant decrease in culturable GFP-*P. aeruginosa* from whole flies (Fig. 2). However taking into account this amount along with the fact that only 16.7% of the flies shed GFP-*P. aeruginosa* during this time interval (Table 1), we infer that additional processes contributed to the observed loss of bacteria within or from house flies in the 0–12 h interval (Fig. 2). Notably, the data presented in Table 1 are an under-representation of both the amount of GFP-*P. aeruginosa* that were excreted and the incidence of excretion events, since long collection intervals subjected bacteria to drying (thus, rendered unrecoverable) and culture plates outside the countable range were not reported (e.g., too few or too many CFUs). Nonetheless, taken together, the excreta and culture recovery experiments suggest that factors other than excretion were important in eliminating and controlling GFP-*P. aeruginosa* numbers within the fly. Therefore, we explored the activity of antimicrobial peptide expression during house fly-bacteria interactions.

**Table 1:** House flies excreted viable GFP-*P. aeruginosa* during multiple time-intervals after ingestion.

Time Interval (h)	No. Flies ThatExcreted (%)	Mean CFU (Range)	
0–2	2 (16.7)		2.50×10^3^ (1140–2710)
0–6	3 (25)		9.52×10^3^ (490–26600)
0–10	2 (16.7)		5.21×10^3^ (520–9900)
0–12	3 (16.7)		9.98×10^4^ (1290–203000)
0–24	5 (41.7)		7.36×10^3^ (470–16400)

Flies were fed a mean of 2.02×10^5^ (SD = 6.30×10^4^) CFU of GFP-*P. aeruginosa.* Incidence of excretion (%) is for n = 12 flies per time interval, and mean CFU is calculated only from flies that excreted viable, countable bacteria.

### House Flies that Ingested GFP-*P. Aeruginosa* Expressed Antimicrobial Peptide Genes both Systemically and Locally

**Figure 3:**
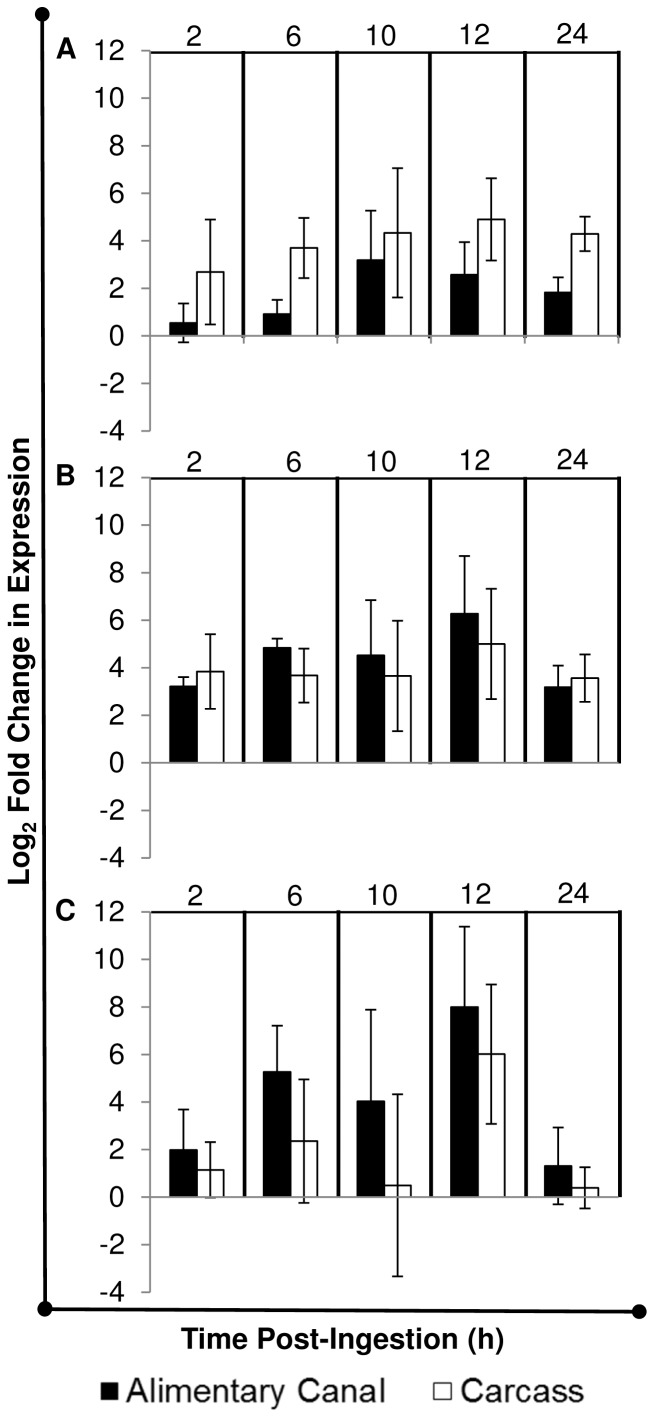
Systemic and local antimicrobial peptide gene expression in house flies that ingested GFP-*P. aeruginosa*. House flies (n = 25 in each of three replicates) were fed an average 3.41×10^5^ CFU (SD = 5.77×10^3^) of GFP-*P. aeruginosa* and at 2, 6, 10, 12, and 24 h post-ingestion, flies (n = 5 per replicate) were dissected to separate the digestive tracts and carcasses which were pooled within replicate/time point for qRT-PCR analysis. Fold changes *cecropin* (A), *defensin* (B), and *diptericin* (C) expression were calculated using the REST-MCS© software by calibrating to AMP expression levels in broth-fed adult flies and using the reference gene *rps18*. Mean log_2_-fold changes in expression are shown, and error bars are standard error. Refer to Table S2 for REST-MCS analysis of systemic and local AMP expression for each replicate.

To understand the fly antimicrobial response after ingestion of GFP-*P. aeruginosa*, we examined systemic (carcass) and local (gut) expression of three AMPs, *cec*, *dpt*, and *def*, whose expression is upregulated after house flies ingest bacteria (Nayduch et al., in revision). House flies expressed all three AMPs in both the gut and carcass as early as 2 h after ingesting GFP-*P. aeruginosa,* and expression was sustained throughout the rest of the observation period (Fig. 3; Table S2). Since GFP-*P. aeruginosa* are confined to the PM in the house fly gut (Fig. 1), it is unclear how or why systemic AMP responses were induced. Systemic AMP expression in the absence of bacteria in the hemolymph could be mediated by communication between the midgut epithelium and fat body, as we have previously postulated in house flies [24]. Possible mechanisms of communication between local and systemic responses have been described in other flies such as tsetse and fruit flies, and include signaling molecules like NO and H_2_O_2_ or diffusing peptidoglycan, respectively [31], [32]. Interestingly, *P. aeruginosa* suppresses AMP expression in *Drosophila* [33], but this was attributable to bacteria escaping the PM and causing systemic infections. Thus, in house flies, the inability of *P. aeruginosa* to traverse the PM may confer protection against hemolymph invasion and prevent subsequent immunosuppression.

Previous studies have demonstrated that short-term starvation of *Drosophila* leads to increased NO production in immune-deficient flies and upregulated AMP expression in bacteria-challenged wild-type flies [34]. In our study, house flies were fed a nutrient-rich meal prior to starvation and were not allowed to feed after ingestion of GFP-*P. aeruginosa* for the rest of the observation period. Although starvation could have altered the immune repertoire of the flies, we utilized this approach to (1) avoid increasing or inducing the rate of peristalsis, which may have caused bacterial elimination and (2) not provide the bacteria with additional nutrients after being ingested, which may have influenced their population dynamics. Future studies should incorporate more realistic conditions where flies ingest a bolus of bacteria, yet are able to feed *ad libitum* on food as they would in nature.

### House Flies Fed GFP-*P. Aeruginosa* Expressed Antimicrobial Peptide mRNA and Protein Regionally in the Alimentary Canal

**Figure 4:**
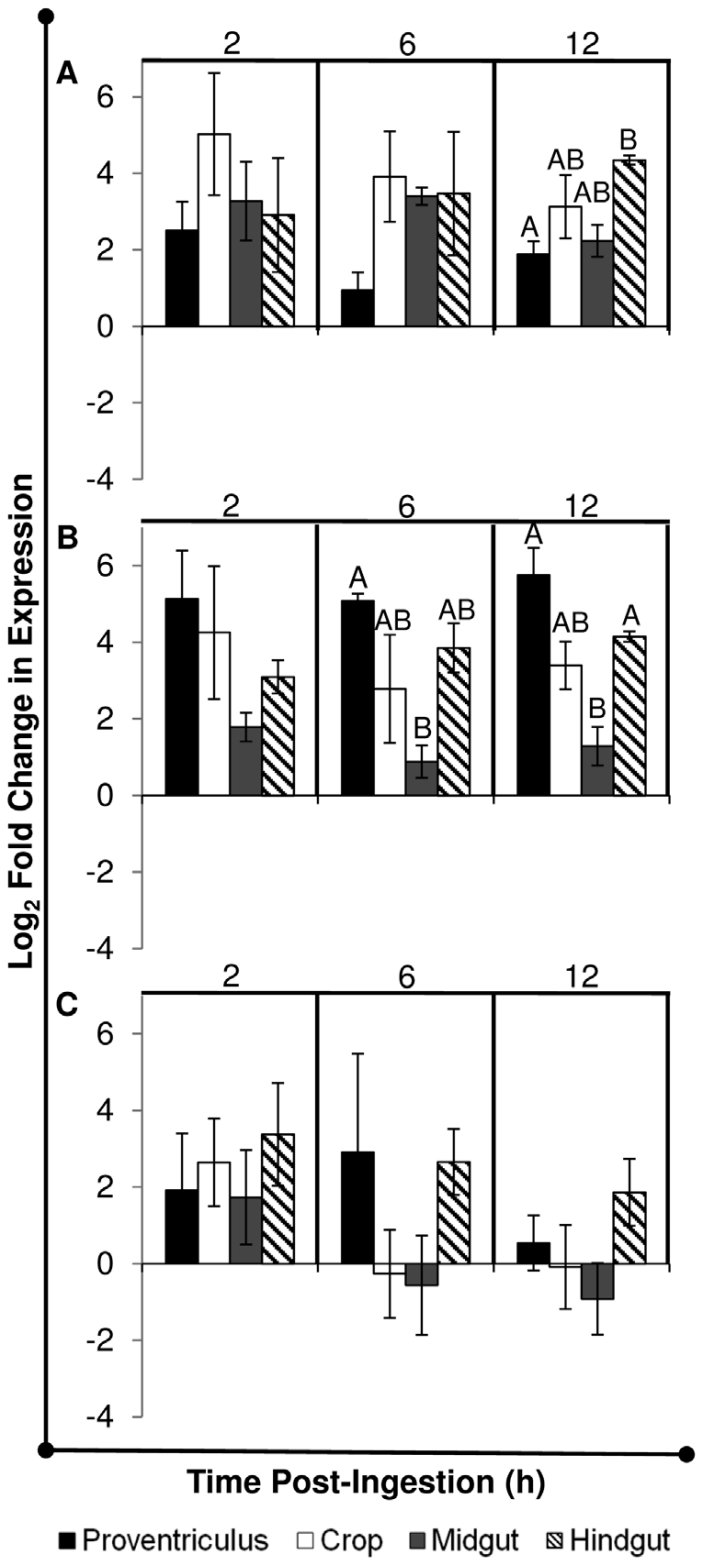
Tissue-specific expression of antimicrobial peptide genes in the alimentary canal of flies that ingested GFP-*P. aeruginosa*. House flies (n = 30 in each of two replicates) were fed an average 6.6×10^4^ CFU GFP-*P. aeruginosa* and at 2, 6, 10, 12, and 24 h post-ingestion, flies (n = 5 per replicate) were dissected to separate the alimentary canal tissues (proventriculus, crop, midgut, hindgut), which were pooled within replicate/time point for qRT-PCR analysis. Fold changes *cecropin* (A), *defensin* (B), and *diptericin* (C) expression were calculated using the REST-MCS© by calibrating to AMP expression levels in unfed adult flies and using the reference gene *rps18*. Mean log_2_-fold changes in expression are shown, and error bars are standard error. Different letters represent significant differences between mean AMP expression levels across tissues within the indicated time point (P≤0.05). Refer to Table S3 for REST-MCS analysis of tissue-specific AMP expression for each replicate.

In addition to local and systemic AMP expression analyses above, the temporospatial expression of AMPs in the alimentary canal was assessed via qRTPCR of mRNA from specific gut tissues (proventriculus, crop, midgut, hindgut/rectum) and immunofluorescent detection of AMP protein in whole gut sections. The aims were to (1) delineate tissue-specific AMP expression profiles and (2) to determine a possible correlation between AMP expression and location of bacteria in the gut. At 2 h PI, *cec*, *dpt*, and *def* were expressed at similar levels across all alimentary canal tissues (Fig. 4; Table S3), which correlated with the presence of bacteria throughout these regions in most flies examined by microscopy (Fig. 1). However *def* was differentially expressed between the proventriculus and midgut at 6 h PI, and proventriculus, hindgut and midgut at 12 h PI (Fig. 4, B; P≤0.05). In addition, *cec* was differentially expressed between the hindgut and proventriculus at 12 h PI (Fig. 4, A; P≤0.05 ). Considering these results, along with microscopy (Fig. 1) and culture (Fig. 2), the differential temporal and spatial expression of *cec* and *def* in the house fly alimentary canal may be attributable to both bacteria location and population density. A homeostatic feedback mechanism between microbe detection and immune induction has not yet been elucidated in house flies, but in *D. melanogaster*, this involves the interplay between peptidoglycan recognition proteins (PGRPs) and bacterial peptidoglycan (PGN) [35]. Fly PGRPs are either (1) membrane bound, for example on gut epithelial cells where they bind bacterial PGN and signal transduction ensues, resulting in AMP synthesis or (2) secreted, where catalytic amidase PGRPs scavenge and cleave PGN into non-stimulatory molecules, thereby preventing nonessential immune induction when bacterial population densities are low and presumably non-threatening. Thus, fruit fly AMPs are produced only when bacteria populations reach a density where the amount of immunostimulatory PGN exceeds the activity of amidase PGRPs in the gut. These PGN molecules traverse the PM, bind transmembrane PGRPs on gut epithelia, and induce AMP production which subsequently reduces the bacterial population [36], [37]. By utilizing amidase PGRPs or other immune-regulatory mechanisms [19], [38], the house fly could efficiently control and/or eliminate ingested microbes while conserving resources by not producing AMPs unnecessarily.

**Figure 5:**
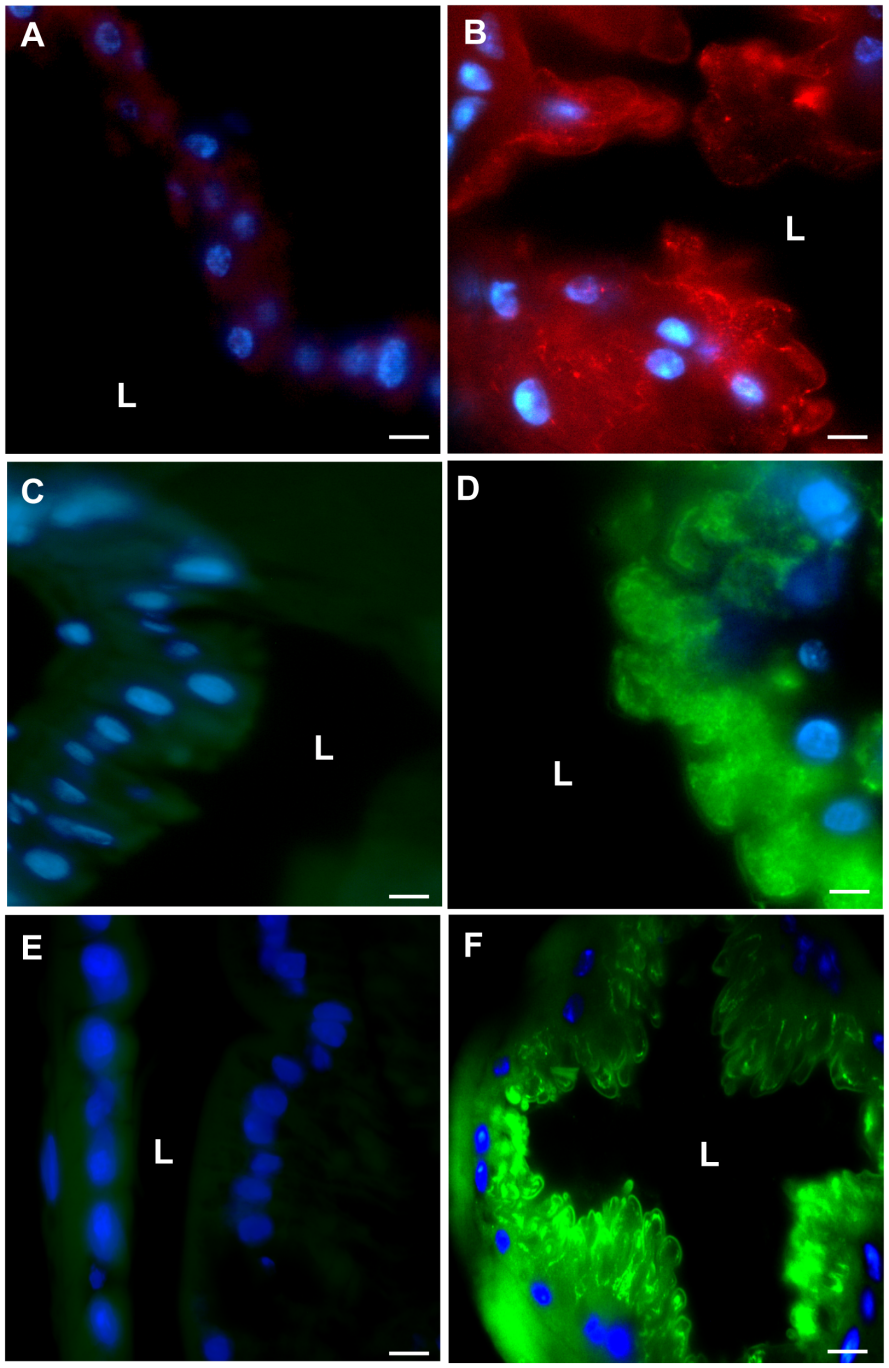
Temporospatial antimicrobial peptide detection in the alimentary canal of house flies fed GFP-*P. aeruginosa*. Flies (n = 12 in each of three replicates) were fed an average of 1.05×10^5^ CFU (SD = 3.37×10^4^) bacteria, and at 4, 6, and 8 h post-ingestion flies (n = 4 per replicate) were dissected to remove the alimentary canal. Control flies (n = 4) were fed sterile BHI broth and dissected at 2 h PI. Immunofluorescent microscopy was used to detect the antimicrobial peptides (AMPs) Cecropin (A, B), Defensin (C, D) and Diptericin (E,F). AMPs were detected in the midgut in flies fed bacteria at 6 h PI (B, D, F) by immunofluorescent microscopy, but not in control flies (A, C, E). AMPs also were detected in the midgut at 8 h PI, and in other tissues (not shown, discussed in the text). L, gut lumen. Scale bar = 10 µm.

We further investigated temporospatial AMP synthesis in the gut of house flies fed GFP-*P. aeruginosa* using immunofluorescent microscopy (Fig. 5). Cecropin, Defensin, and Diptericin were detected in midgut cells at 6 h (Fig. 5, B, D, F) and 8 h PI (data not shown), which correlated with both the predominant location of GFP-*P. aeruginosa* in the digestive tract (Fig. 1) and coincided with the observed decrease in recoverable bacteria from flies (Fig. 2). Cecropin and Diptericin expression varied between flies, as well as biological replicates, after ingestion of GFP-*P. aeruginosa*; however, these AMPs were ephemerally expressed in multiple alimentary canal regions including proventriculus and crop in some flies (data not shown). Notably, these two AMPs were consistently expressed in the midgut at 6 and 8 h PI (Fig. 5 B, D; data not shown). In contrast, Defensin was consistently upregulated in all flies that ingested GFP-*P. aeruginosa* across all replicates and in all regions on the alimentary canal that were examined (crop, proventriculus, midgut, hindgut) at 4, 6, and 8 h PI (midgut shown in Fig. 5; other organs, data not shown). While we did not examine the activity of Defensin against *P. aeruginosa* in this study, house fly Defensin previously has been shown to have a wide spectrum of activity [39] and is the primary AMP that is induced after feeding a wide variety bacteria species, irrespective of PGN type al., [24;Nayduch et al., in revision]. We speculate that this AMP, along with other antimicrobial processes such as sequestration of bacteria in the PM, are key factors influencing fly-microbe dynamics, and subsequently bacteria fate, persistence, and transmission potential.

## Conclusions

To gain insight into how fly-microbe interactions ultimately impact vector competence, we monitored the fate of bacteria in house flies along with the concurrent temporospatial antimicrobial response. We utilized a GFP-expressing strain of *P. aeruginosa* that facilitated visualization and culture recovery, and allowed for qualitative and quantitative assessments of the microbial population dynamics of this organism after being ingested by house flies. Like other species of bacteria, *P. aeruginosa* was enclosed in the inner PM in the midgut, and was unable to colonize the epithelium or invade the house fly. We surmise that the PM serves as a primary and effective physical barrier to ingested bacteria in the house fly midgut.

Although we observed a significant decrease in the number of recoverable GFP-*P. aeruginosa* in house flies around 12 h post-ingestion, bacteria populations recovered and apparently proliferated to reach levels near the original dose that was fed. GFP-*P. aeruginosa* remained motile in the house fly gut, and we speculate that this allowed bacteria to avoid peristalsis and also to move away from unfavorable, hostile areas of the gut (e.g. extreme pH or ionic conditions, digestive enzyme activity, antimicrobial peptides) to areas where populations could presumably recover and proliferate. The exact role that motility serves in bacterial survival in house flies deserves further study, as many non-motile bacteria or immobilized strains of motile bacteria (discussed above) consistently seem to be rapidly lysed and excreted from house flies once they enter the midgut.

Flies that ingested GFP-*P. aeruginosa* expressed AMPs both locally in the gut and systemically in the carcass. While the activity of these AMPs against *P. aeruginosa* was not investigated, AMP protein expression in the midgut, where we bacteria were observed, coincided with a decrease in the number of bacteria recovered from flies. We did not determine whether AMP protein production ceases when bacteria levels drop, and it would be intriguing to measure the long term fluctuations and feedback between bacteria population counts and AMP protein-level expression. Other AMPs and antimicrobial effectors likely play a role in house fly-microbe dynamics and warrant further study. Additionally, the underlying feedback mechanisms between fly immune responses and bacteria population densities still remain unknown, and we know very little about gut-microbe interactions in higher diptera except for the model organism *D. melanogaster.* Since the outcome of these dynamic interactions impacts bacterial persistence, survival and vector competence, and because different bacterial species have different fates within house flies, we can conclude that house flies do not have uniform vector competence across bacteria species. Future studies assessing the molecular and microbiological interactions between house flies and other microbe species will help us understand the role these interactions play in vector competence for pathogens and may serve as a platform for designing novel interventions for fly-transmitted diseases.

## Supporting Information

Figure S1 **Viable GFP-** ***P. aeruginosa*** **in the crop and rectum of the house fly suggests both oral and fecal transmission of bacteria.** House flies (n = 20 in each of three replicates) were fed an average of 2.12×10^5^ CFU (SD = 8.36×10^4^) bacteria and at 2, 6, 10, 12, and 24 h post-ingestion (PI), flies (n = 4 per replicate) were dissected to obtain intact alimentary canals for epifluorescent microscopy. GFP-expressing bacteria (green rods, arrows) were seen in the crop and rectum at all time points. A and B, representative images of the crop at 6 and 24 h PI, respectively. C, bright field image of rectum with fecal material at 10 h PI (blue box); D is an epifluorescent view of the fecal material (blue box, C) showing viable bacteria (arrows). Scale bars: A, B, and D = 10 µm and C = 100 µm.
(TIF)

Table S1 **Primer sequences used for qRT-PCR analyses of antimicrobial peptide gene expression in house flies fed GFP-** ***P. aeruginosa*** **.**
(DOCX)

Table S2 **Systemic and local antimicrobial peptide expression analysis using REST-MCS©.** House flies (n = 25 in each of three replicates) were fed an average of 3.41×10^5^ CFU (SD = 5.77×10^3^) of GFP-*P. aeruginosa* and processed as described in the text. A pairwise fixed allocation randomization test was performed using REST-MCS® to analyze AMP gene expression. P-values are for comparison to the calibrator state using the reference gene *rps18*. Statistically significant P-values are shown in yellow. Red and blue represent upregulation and downregulation of target genes, respectively.
(XLSX)

Table S3 **Tissue-specific antimicrobial peptide expression analysis using REST-MCS©.** House flies (n = 30 in each of two replicates) were fed an average of 6.6×10^4^ CFU GFP-*P. aeruginosa* and processed as described in the text. A pairwise fixed allocation randomization test was performed using REST-MCS® to analyze AMP gene expression. P-values are for comparison to the calibrator state using the reference gene *rps18*. Statistically significant P-values are shown in yellow. Red and blue represent upregulation and downregulation of target genes, respectively.
(XLSX)
