# Supplementary figures and images for: Pseudomonas aeruginosa in Musca domestica L.: Temporospatial Examination of Bacteria Population Dynamics and House Fly Antimicrobial Responses
Source: PLoS One. 2013 Nov 18;8(11):e79224. doi: 10.1371/journal.pone.0079224 (PMC3832466; doi:10.1371/journal.pone.0079224)

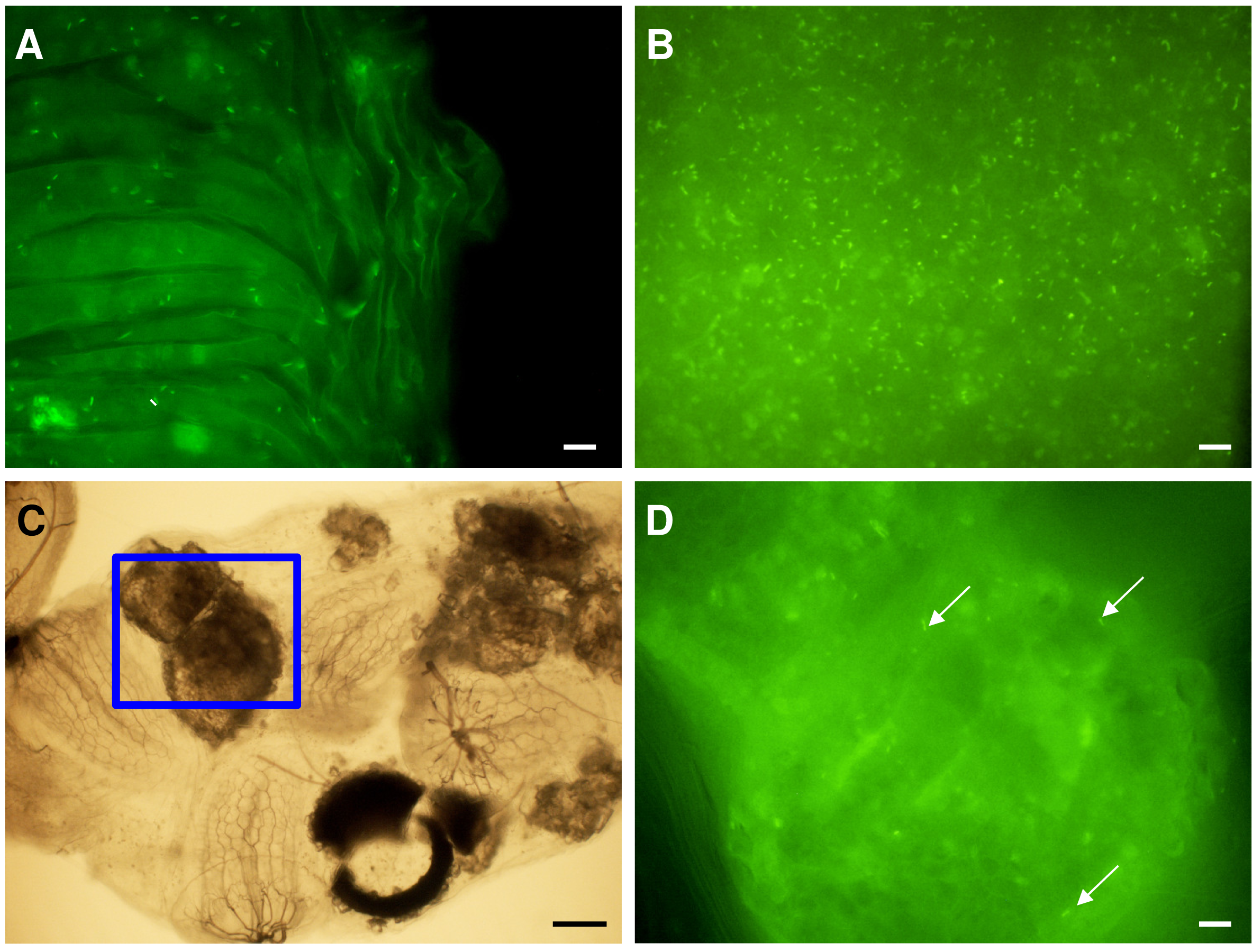

Supplement: Figure S1 — Viable GFP- P. aeruginosa in the crop and rectum of the house fly suggests both oral and fecal transmission of bacteria. House flies (n = 20 in each of three replicates) were fed an average of 2.12×10^5^ CFU (SD = 8.36×10^4^) bacteria and at 2, 6, 10, 12, and 24 h post-ingestion (PI), flies (n = 4 per replicate) were dissected to obtain intact alimentary canals for epifluorescent microscopy. GFP-expressing bacteria (green rods, arrows) were seen in the crop and rectum at all time points. A and B, representative images of the crop at 6 and 24 h PI, respectively. C, bright field image of rectum with fecal material at 10 h PI (blue box); D is an epifluorescent view of the fecal material (blue box, C) showing viable bacteria (arrows). Scale bars: A, B, and D = 10 µm and C = 100 µm. (TIF) [file pone.0079224.s001.tif]
