# Supplementary material for: Pseudomonas aeruginosa in Musca domestica L.: Temporospatial Examination of Bacteria Population Dynamics and House Fly Antimicrobial Responses
Source: PLoS One. 2013 Nov 18;8(11):e79224. doi: 10.1371/journal.pone.0079224 (PMC3832466; doi:10.1371/journal.pone.0079224)
Supplement: Table S1 — Primer sequences used for qRT-PCR analyses of antimicrobial peptide gene expression in house flies fed GFP- P. aeruginosa . (DOCX) [file pone.0079224.s002.docx]

**Table S1. qRT-PCR primer sequences used for antimicrobial peptide gene expression analyses of house flies fed GFP-*P. aeruginosa***.

| **Gene** | **Acc. no.** | **Primer sequence** |
| --- | --- | --- |
| *cecropin* | GABS01000319 | Fwd: 5̍-GGACAAAGTGAAGCTGGCTGGTTA-3̍ |
|  |  | Rev: 5̍-GCTGGGCCACACCAATAGTTTGAA-3̍ |
| *defensin* | GABS01000169 | Fwd: 5̍-AAATTTCGTCCATGGAGCTGACGC-3̍ |
|  |  | Rev: 5̍-ACCGCTCAACAAATCGCAAGTAGC-3̍ |
| *diptericin* | GABS01000119 | Fwd: 5̍-AGTGCAACATTTGTGGTTGCCGAC-3̍ |
|  |  | Rev: 5̍-GCCATAACCTGCTGTGGCATCAAA-3̍ |
| *rps18* | GABS01000063 | Fwd: 5̍-GTTGGTATTGCCATGACCGCCATT-3̍ |
|  |  | Rev: 5̍-ATGGGTTGGAGATGATGGTGACGA-3̍ |
